# A Meta-Analytic Review of Moral Disengagement and Cyberbullying

**DOI:** 10.3389/fpsyg.2021.681299

**Published:** 2021-11-30

**Authors:** Lijun Zhao, Junjian Yu

**Affiliations:** Department of Psychology, School of Education Science, Liaocheng University, Liaocheng, China

**Keywords:** moral disengagement, cyberbullying, meta-analysis, moderating effect, cross-culture

## Abstract

With the development of technology, cyberbullying prevalence rates are increasing worldwide, and a growing body of the literature has begun to document cyberbullying behavior. Moral disengagement is often considered a key correlate factor in cyberbullying. This article aims to conduct a meta-analysis review of the relationship between moral disengagement and cyberbullying and some psychosocial and cultural variables. Based on the PRISMA method, a random-effects meta-analysis is employed in this study to obtain reliable estimates of effect sizes and examine a range of moderators (age, gender, measure method, and cultural background). Relevant studies, published from 2005 to February 30, 2021, were identified through a systematic search of the Web of Science, ScienceDirect, SpringerLink, Pubmed, EBSCO, and Wiley Online Library. Finally, 38 studies (*N*=38,425) met the inclusion criteria. The meta-analysis conclusion demonstrated that moral disengagement positively correlated medium intensity with cyberbullying (*r*=0.341). Age, gender, and cultural background had moderated the relationship between moral disengagement and cyberbullying.

## Introduction

With the development of science and technology, Internet communication technology has been continuously popularized and applied. Meanwhile, mobile phones, computers, and other network communication devices have become an indispensable part of people’s daily life. According to the 2021 Internet World Statistics, the number of global Internet users has reached 5.16 billion as of March 31, 2021. As the number of Internet users is growing, cyberbullying based on online media is also increasing year by year, and it has become a vital issue of concern worldwide (Gini et al., 2014a; Lee and Shin, 2017; Kowalski et al., 2018; Zhu et al., 2021).

Cyberbullying is typically defined as online aggression behavior, which is intentionally and repeatedly conducted in an electronic platform (e.g., email, blogs, instant messages, and text messages) against a person who cannot easily defend him or herself (Kowalski et al., 2014; Chan et al., 2021). The main forms of cyberbullying include online abuse, online intimidation, online isolation, disclosure of privacy, and online disguised identity (Menesini and Spiel, 2012; Zych et al., 2019a). The media where cyberbullying occurs are also diverse, including instant messaging, emails, web pages, chat rooms, social networking sites, digital images, and online games (Kowalski et al., 2018; Paciello et al., 2020). What’s more, recent meta-analyses indicated that the prevalence of cyberbullying among school-age children ranges from 13.99 to 57.5% (Bussey et al., 2015a; Zhu et al., 2021), while the incidence of cyberbullying among young adults range from 8 to 28% (Francisco et al., 2015; Selkie et al., 2015; Kowalski et al., 2018).

Cyberbullying has the characteristics of anonymity, virtuality, and concealment. Compared with face-to-face bullying, cyberbullying occurs in the online environment, in which victims cannot quickly recognize the identity of the cyberbullies (Gini et al., 2014a; Wang and Ngai, 2020; Zhu et al., 2021). Perpetrators of cyberbullying often perceive themselves to be anonymous (Wang and Ngai, 2020). Most cyberbullying messages are sent in the form of nicknames, generating an opportunity for cyberbullies to hide (Wang et al., 2016; Yang et al., 2018). Moreover, individuals can observe the influence of their behavior on the victim in face-to-face bullying. However, the virtual environment of cyberspace makes it impossible for cyberbullying perpetrators to have a direct way to understand the impact of their behavior on the victim (Sourander et al., 2010; Kowalski et al., 2014). For some perpetrators, the awareness that they have hurt the victim is enough to prevent further bullying. Cyberbullying is not restricted by time and space and is more likely to cause severe physical and psychological harm to individuals (Kowalski et al., 2014, 2018). Cyberbullying can lead to undesirable behaviors and health-related issues, resulting in depression, anxiety, stress, and adverse emotional problems. Moreover, it can lead to suicide problems in extreme cases (Lam and Li, 2013; Kowalski et al., 2014, 2018).

## Moral Disengagement and Cyberbullying

Firstly, Bandura et al. (1996) proposed the concept of “Moral Disengagement” based on social cognition theory. It refers to helping individuals redefine their cognitive-behavioral tendencies, thus making them feel less guilt and shame to victims. This explains why people do not feel pain and self-accusation even when they commit cruel acts of harm. Moral disengagement is a cognitive mechanism, which can be divided into eight mechanisms: moral defense mechanism, euphemistic labeling mechanism, responsibility transfer mechanism, favorable comparison mechanism, responsibility dispersion mechanism, result in distortion mechanism, dehumanization mechanism, and blame attribution mechanism (Bauman, 2010; Meter and Bauman, 2016). Moral disengagement is an important cognitive basis for the generation of individual aggression (Gini et al., 2014a; Kowalski et al., 2014; Zych et al., 2019a). Several studies have shown a strong connection between moral disengagement and bullying behaviors (Gini et al., 2014a; Kowalski et al., 2014; Zych et al., 2019a; Gaffney et al., 2019). Individuals can redefine their bullying behavior through the moral disengagement mechanism. It is an effective predictor of aggression and cyberbullying behavior (Bandura et al., 1996; Pornari and Pornari, 2010; Kokkinos et al., 2016; Luo and Bussey, 2019). For example, to avoid their negative self-evaluation and shame (Bauman, 2010; Meter and Bauman, 2016), they consider that their cyberbullying actions are less harmful to the victim and the victim should be punished.

The effect of moral disengagement on traditional bullying is clear (Paciello et al., 2020; Romera et al., 2021a; Travlos et al., 2021), while the relationship between moral disengagement and cyberbullying remains controversial (Lo Cricchio et al., 2021). Firstly, the characteristics of offline and online moral disengagement and cyberbullying are different. Individuals engaging in cyberbullying can perpetrate cyberbullying behavior 24h a day, 7days a week. During the day or night, they can create websites, send messages, or post pictures about others on the Internet at any time (Kowalski et al., 2014, 2018). Traditional bullying occurs most frequently face to face during school days (Paciello et al., 2020). Cyberbullying is not limited by time or place, as it may occur at any time and can reach the victim anywhere. Cyberbullying material is shared online. It is easy for people to share, retweet, and repeat bullying messages.

These materials are hard to remove so that the bullying can last for a long time (Wang et al., 2009; Zych et al., 2019b). Thousands of people may view insulting posts online, while only several may view bullying incidents at school (Menesini and Spiel, 2012; Paciello et al., 2020). Cyberbullying, which has a much greater potential audience than traditional bullying, has a more severe impact on victims (Martínez et al., 2019; Paciello et al., 2020). Compared to offline, individuals with lower moral levels are more likely to engage online and engage in cyberbullying (Perren and Gutzwiller-Helfenfinger, 2012; Orue and Calvete, 2019; Romera et al., 2021b).

Secondly, the influence mechanism of moral disengagement on traditional bullying and cyberbullying is different. Cyberspace is invisibility, publicity, and shareability, which does not have space and time boundaries (Wang and Ngai, 2020). In such a virtual network society, individuals can ignore the social norms and social pressures from the real world. Thus, their cyberbullying behavior is more likely to be associated with a higher level of moral disengagement (Postmes and Spears, 1998; Busching and Krahé, 2015).

The virtual online world seems to be characterized by a degree of disinhibition (Suler, 2004; Wright, 2014), which is a crucial social environment for moral disengagement (Bandura et al., 1996; Bauman, 2010; Meter and Bauman, 2016). At the same time, under the conformity and the accessibility of cyberspace, cyberbullying is increasingly being used as an emotional outlet by more and more people (Gini et al., 2018). Moreover, the social media environment might accelerate the emergence of moral disengagements, such as diffusion of responsibility, blame attribution of the victims, and result in distortion (Meter and Bauman, 2016). In this case, individuals can freely explain their behavior to defend themselves (Runions and Bak, 2015; Kowalski et al., 2018). The virtual online world lacks social norms, supervision mechanisms, and moral evaluation systems. Therefore, it is difficult for people to form a “heterogeneous morality” influenced by external norms. These aspects, in turn, can increase the likelihood of individuals engaging in cyberbullying behaviors.

Additionally, although there were some sporadic studies on the effect of moral disengagement on cyberbullying, its effect sizes were inconsistent across different studies. For example, Lazuras et al. (2019) measured the correlation coefficient between moral disengagement and cyberbullying in Greek and Italian participants, which was −0.150 and 0.35, respectively. Wang et al. (2016) calculated the correlation coefficient between moral disengagement and cyberbullying, 0.52 and 0.28 for male and female participants, respectively. The correlation coefficient between moral disengagement and cyberbullying was 0.16 and 0.47 in Meter and Bauman (2016) and Bussey et al. (2020), respectively.

Therefore, it is necessary to integrate a large number of relevant works of the literature to explore the relationship between moral disengagement and cyberbullying.

## Moderators Between Moral Disengagement and Cyberbullying

To examine the meta-analysis relationship between moral disengagement and cyberbullying, we also examined whether these relationships varied depending on moral disengagement measuring tools, age, gender, and cultural background.

Regarding measuring tools, different research tools may have different impacts on the relationship between moral disengagement and cyberbullying. The original scale of moral disengagement was a 32-item scale developed by Bandura et al. (1996), which was used to measure the degree of moral disengagement, including eight moral disengagement mechanisms. The items were assessed using a five-point Likert Scale ranging from strongly disagree to strongly agree. This scale has been widely used in Chinese samples and has good reliability and validity (Wang et al., 2019c, 2020). Adolescent Version of Moral Disengagement Scale (MDS; Bandura et al., 1996) was used to assess the acceptance of moral exemption for harmful conduct. The scale consists of 24 items to evaluate six moral disengagement mechanisms, including moral justification, advantageous comparison, distorting consequences, displacement of responsibility, diffusion of responsibility, and attribution of blame. Pelton et al. (2004) tested the structure, reliability, and correlation of the MDS (Bandura et al., 1996) in the United States. The study found that MDS has similar factor structures, internal consistency, and demographic results in the US participants. Furthermore, the role of moral disengagement in the correlation between parenting and child behavior was examined. Gini et al. (2014b) developed Classroom Collective Moral Disengagement Scale for adolescents, which refers to shared group beliefs that morally justify negative actions. It is promising that the scale is a measure for research concerning group-level morality. Ribeaud and Eisner (2010) conducted related research on the items in the MDSs, and the results suggested a correlation between them (*r*=0.51). Some researchers revealed a large degree of overlap in the concept of items measured by different scales. Related research demonstrated that the relationship between moral disengagement and cyberbullying is affected by the common method bias (Gini et al., 2014a). The effect of measuring tools on the relationship between moral disengagement and cyberbullying is uncertain. Therefore, different moral disengagement measuring scales on the relationship between them should be investigated.

Regarding age, the purpose of measuring this moderator was to explore the changes in the relationship between moral disengagement and cyberbullying behavior across different ages. At present, some researchers have reported age differences between them, while the research participants were mainly focused on adolescents and children (Gini et al., 2014a; Kowalski et al., 2018; Zhu et al., 2021). As well, the studies on cyberbullying indicated that the rate of cyberbullying among adolescents was higher than that among children. Robson and Witenberg (2013) suggested that age had a significant predictive effect on the participation of cyberbullying. The proportion of 14–15years old students participating in cyberbullying was higher than that of 12–13years old students. Gini et al. (2014a) discovered that teenagers (12–18years old) had higher levels of cyberbullying than children (8–11years old). A longitudinal study on the Internet bullying behavior of German teenagers also revealed that cyberbullying behavior gradually increased with the growth of age (Scharkow et al., 2014). Adolescent cyberbullying was more common in high school (Álvarez-García et al., 2018; Calmaestra et al., 2020). Nevertheless, some relevant studies pointed out that there was little analysis and research on the cyberbullying behavior of adult participants, and the cyberbullying behavior of adult participants needs to be deeply explored (Chan et al., 2021). Therefore, we propose the hypothesis that age has a significant moderating effect on the relationship between moral disengagement and cyberbullying. The degree of cyberbullying of adult participants between moral disengagement and cyberbullying is higher than that of adolescent participants.

In previous studies, the issue of the gender between moral disengagement and cyberbullying has always been the focus of scholars’ research, with three completely different views. Firstly, there is no connection in gender between moral disengagement and cyberbullying. They considered that cyberbullying happened in cyberspace is similar (Wang et al., 2019a), and there is no difference in gender, so the correlation between moral disengagement and aggressive behavior is not significantly different between boys and girls (Lipsey and Wilson, 2001; Martinez-Pecino and Durán, 2019; Marr and Duell, 2020). Nevertheless, some researchers consider that there are gender differences in a specific form of cyberbullying. For example, girls usually use emails or chat rooms for cyberbullying (Zych et al., 2019a), while boys often employ text messages or online games for cyberbullying (Wang et al., 2016; Romera et al., 2021a). They thought that individuals of different genders have different preferences for bullying behavior (Kowalski et al., 2018). Secondly, the relationship between moral disengagement and cyberbullying was stronger for females than for males (Kowalski and Limber, 2007; Kowalski et al., 2014; Marcum et al., 2014). The results of the meta-analysis by Kowalski et al. (2014) suggested that gender could significantly moderate the relationship between moral disengagement and cyberbullying. Specifically, the correlation coefficient between moral disengagement and cyberbullying increases as the proportion of women in the sample increases. Girls are prone to hidden aggression. It is reported that girls’ aggression is more covert rather than overt because it uses note-sharing, “hate books,” isolation from peer groups, and various forms of anonymous call (Burnham et al., 2011; Marr and Duell, 2020). Thirdly, the relationship between moral disengagement and cyberbullying was stronger for males than that for females (Erdur-Baker and Kavsut, 2010; Wang et al., 2016; Calmaestra et al., 2020; Gao et al., 2020). Boys show fewer moral feelings (e.g., guilt and empathy) than girls (Bussey et al., 2015b), who are a lower desire for personal relationship building, which would be associated with a greater engagement in cyberbullying. In contrast, girls, they desire positive relations with others may tend to limit their engagement in cyberbullying behaviors, even when they have higher levels of moral disengagement (Samnani et al., 2014; Wang et al., 2016). Based on the above research, it is necessary to explore further the role of gender in the relationship between moral disengagement and cyberbullying. Therefore, we hypothesize that gender has a significant moderating effect on the relationship between moral disengagement and cyberbullying.

Finally, we researched the cultural background. Cross-cultural research on cyberbullying is often reported by researchers. However, there is no comparative analysis of cultural background differences in cyberbullying globally due to the small number of previous studies and the small sample size (Kowalski et al., 2014). Some researchers suggested that cultural differences may be reflected in cyberbullying behaviors under Hofstede’s cross-cultural analysis model. Hofstede divides the cross-cultural model into five dimensions: power distance, long-term orientation index, uncertainty avoidance, masculinity or feminality, and individualism or collectivism. In the 2010 study, he added a sixth dimension: Indulgence versus Restraint. Kowalski et al. (2014) discovered that the relationship between cyberbullying and loneliness, self-esteem, and moral disengagement in the North American samples was higher than that in the European and Australian samples, considering that cyberbullying behavior has differences in individualism/collectivism between North America and other places. Zhu et al. (2021) found that cyberbullying had country differences. The incidence of cyberbullying in the United States of America is 15.5–31.4%, and the incidence in Israel is 30–45%. China has the highest incidence of cyberbullying, ranging from 6 to 46.3%. Canada has the lowest incidence of cyberbullying at 7.99%. These results are related to cultural backgrounds. Shapka and Law (2013) demonstrated that the relationship between moral disengagement and cyberbullying among East Asian teenagers was higher than that among European teenagers. The eastern cultural background belongs to collectivism, while the western culture belongs to individualism. In collectivist cultures, people like doing things together. It is rare for people to be the first to engage in cyberbullying, and they are often not the leaders in cyberbullying incidents. Usually, when someone does this cyberbullying, people will follow suit. Thus, the number of cyberbullies has gradually increased. In collectivist cultures, the individual’s moral disengagement mechanisms are more likely to be activated, and the number of cyberbullies usually exceeds the number of victims because cyberbullies often act in groups (Kowalski et al., 2018; Paciello et al., 2020). Furthermore, group cyberbullying is more common in the collectivist culture. Cyberbullying generally happens among peer groups, rarely one-on-one (Cassidy et al., 2013; Killer et al., 2019). Therefore, we hypothesize that cultural background has a significant moderating effect on the relationship between moral disengagement and cyberbullying.

## The Current Research

Gini et al. (2014a) conducted the first meta-analysis of the relationship between moral disengagement and cyberbullying. However, only four research samples of moral disengagement and cyberbullying were included, composed of only children and adolescents. Wang et al. (2014) meta-analyzed moral disengagement and cyberbullying, and only three sample data on cyberbullying were included. Kowalski et al. (2014) conducted a meta-analysis of the relationship between cyberbullying and cyberbullying victims, self-esteem, compassion, substance abuse, life satisfaction, school security, anger, loneliness, and academic achievement, with only seven data samples, revealing that the correlation coefficient between moral disengagement and cyberbullying was 0.27. The subjects of this study are mostly children and adolescents under the age of 18, making it not comprehensive enough. Moderating effect analysis was not performed owing to the limitation of small samples at that time. However, Kowalski et al. (2018) discovered differences in the prevalence of cyberbullying among different age groups, such as children, adolescents, and adults. Research on adulthood is notably lacking. More research is needed to investigate the effect of age on cyberbullies. Zhu et al. (2021) considered that cyberbullying has age, gender, and regional cultural differences, while the reasons for these differences need to be further explored.

In the past, most studies analyze various variables, such as moral disengagement, empathy, and depression on cyberbullying, resulting in an insufficient sample size for presenting the specific impact of moral disengagement on cyberbullying. Therefore, in this study, the relationship between moral disengagement and cyberbullying is mainly analyzed, as well as adult samples in our research, to compare the differences between adult and adolescent participants in the relationship between moral disengagement and cyberbullying.

This meta-analysis attempts to solve two major questions. (1) What is the effect size of the correlation coefficient between moral disengagement and cyberbullying? (2) Whether measuring tools, age, gender, and cultural background affect the relationship between moral disengagement and cyberbullying? This study aims to cover the latest research and the most extensive database. To explore the relationship between moral disengagement and cyberbullying, we searched published papers from 2005 to February 2021 on several databases. The measuring tools, gender, age, and cultural background were to be analyzed as a moderating variable.

## Materials and Methods

### Search Strategy

The systematic literature search strategy is based on the PRISMA statement (Moher et al., 2009; Yap and Jorm, 2015). Papers were searched in several electronic databases, including the Web of Science, ScienceDirect, SpringerLink, Pubmed, and Wiley Online Library. A wide range of search terms was provided to ensure that the included articles were comprehensive and specific. Relevant studies contained at least one keyword in the title, abstract, and/or keywords from each of the two aspects: moral disengagement and cyberbullying (see Table 1). Wildcards and logical operators were adopted to minimize the number of missed documents in database searches by ensuring that we searched the most extensive literature. Moreover, we looked at the contents of major journals in the field and manuallyexamined the citations of highly cited studies on the research issues.

**Table 1:** Keywords of two search aspects.

Search aspects
(A) **Cyberbullying**: cyberbullying OR “*cyberbullying” OR cyberbullying OR cyber aggression OR cyber attack OR online attack OR online bullying OR electronic bulling OR Internet bulling OR “online aggression” OR “electronic aggression” OR “Internet aggression.”
(B) **Moral disengagement**: moral disengagement OR moral evasion OR moral shirk OR moral escape OR “moral disengagement” OR “evasion*” OR “shirk* OR “*escape” OR “moral*.”

Asterisks are wildcards. Wildcards are used in place of one or more real characters associated with cyberbullying and moral disengagement. The aim is to ensure that we can search the widest possible literature.

### Inclusion and Exclusion Criteria

**Figure 1:**
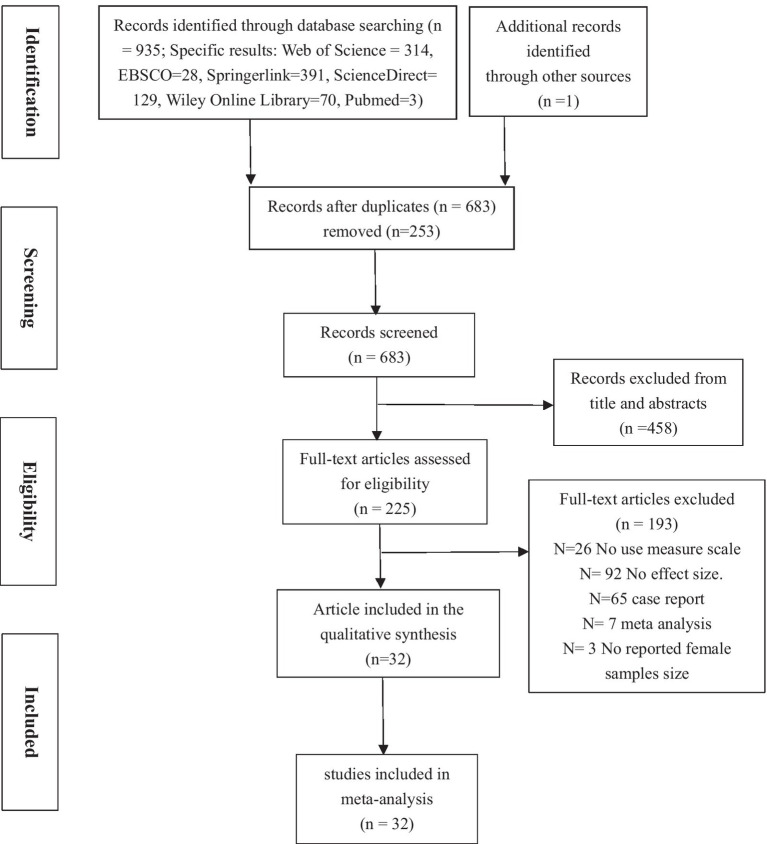
PRISMA flow chart diagram showing the process of study selection for inclusion in the systematic review on moral disengagement and cyberbullying.

The criteria for inclusion and exclusion in this study were described as follows. (1) The research must be empirical research on moral disengagement and cyberbullying. Specific survey data should be reported, and pure theoretical research, such as literature review and field research, would be excluded. (2) The literature must report a clear sample size and the correlation coefficient *r* between moral disengagement and cyberbullying, or other complete data that can be converted into an effect size *r*. (3) The scales used in the literature must be complete and specific, and the MDS and cyberbullying scale must be reported with good reliability and validity. The details of the employed screening process are illustrated in Figure 1, in which a total of 38 studies (*n*=38,425) were included in our final review.

### Coding Procedures

The literature meeting the meta-analysis inclusion criteria were coded as literature information (the first author and publication time), sample size(*N*), effect size(*r*), age of participants (adolescents vs. adults), moral disengagement measurement questionnaire (Bandura original vs. Bandura revision vs. others), cultural background (Collectivism vs. Individualism), and the proportion of female participants. Among them, subjects aged <18years old and≥18years old were coded as adolescents and as adults, respectively. The cultural background was coded as the collectivism or individualism dimension according to Hofstede’s cross-cultural model (Hofstede and Minkov, 2010). China (Regarding Hofstede’s study, the score for individualism is 25. The lower the score, the greater the collectivism; the higher the score, the greater the individualism. The same as below), Arab countries (38), Turkey (37), Greece (35), South Korea (18), Spain (51), and Iran (41) are coded as Collectivism; the United States (91), Australia (90), England (89), Canada (80), Netherlands (80), Italy (76), and Germany (67) are coded as Individualism. The effect size of each independent sample in the literature was coded only once. If literature contained multiple independent samples, they were coded separately; if the effect size of boys and girls was reported independently in the literature, they were separately coded. This produced multiple independent effect sizes. The details are listed in Table 2.

**Table 2:** Study characteristics.

Study author(s) (year)	*N*	*r*	Age/Grade	Sample age group	MD Measure	Culture background	Female(%)
Robson and Witenberg, 2013	210	0.190	13.2±1.1	Adolescents	Bandura original	Individualism	50
Meter and Bauman, 2016	800	0.160	Grade3-8	Adolescents	Others	Individualism	52
Fernández-Antelo and Cuadrado-Gordillo, 2019	1,521	0.410	12.1±1.3	Adolescents	Bandura original	Collectivism	52
Cuadrado-Gordillo and Fernández-Antelo, 2019	1,912	0.430	14–18	Adolescents	Bandura original	Collectivism	51
Orue and Calvete, 2019	765	0.380	14–18	Adolescents	Bandura original	Collectivism	60.65
Orue and Calvete, 2019	765	0.340	14–18	Adolescents	Bandura original	Collectivism	60.65
Fang et al., 2020	650	0.650	18–24	Adults	Bandura revision	Collectivism	64
Lazuras et al., 2019	1,710	−0.150	16.35±1.49	Adolescents	Bandura original	Individualism	54.5
Lazuras et al., 2019	355	0.350	14.76±1.20	Adolescents	Bandura original	Collectivism	55.5
Paciello et al., 2020	856	0.370	14.7±1.7	Adolescents	Bandura original	Individualism	45.6
Hoareau et al., 2020	334	0.290	11–15	Adolescents	Bandura original	Individualism	48.5
Perren and Gutzwiller-Helfenfinger, 2012	495	0.138	11–18	Adolescents	others	Individualism	47
Wang et al., 2019c	412	0.370	13.53±0.91	Adolescents	Bandura revision	Collectivism	53.9
Yang et al., 2018	649	0.260	11–18	Adolescents	Bandura revision	Collectivism	48
Wang et al., 2017	464	0.440	17–25	Adults	Bandura original	Collectivism	65
Wang et al., 2019b	404	0.310	13.53±0.92	Adolescents	Bandura revision	Collectivism	53.22
Ramadan, 2019	140	0.390	15.90±1.03	Adolescents	others	Collectivism	42.86
Bartolo et al., 2019	571	0.340	15.81±1.36	Adolescents	Bandura original	Individualism	45.5
Bauman, 2010	190	0.320	Grade5-8	Adolescents	Bandura original	Individualism	54
Zych et al., 2019a	598	0.322	Grade5-6	Adolescents	Bandura original	Collectivism	46.3
Zych et al., 2019b	885	0.315	Secondary1-4	Adolescents	Bandura original	Collectivism	48.8
Bussey et al., 2020	540	0.470	11–15	Adolescents	Bandura revision	Individualism	56.3
Allison and Bussey, 2017	563	0.340	Grade7-9	Adolescents	Bandura revision	Individualism	39.43
Allison and Bussey, 2017	563	0.230	Grade7-9	Adolescents	Bandura revision	Individualism	39.43
Wang et al., 2016	215	0.520	12–14	Adolescents	Bandura original	Collectivism	0
Wang et al., 2016	202	0.280	12–14	Adolescents	Bandura original	Collectivism	100
Bussey et al., 2015a	964	0.360	Grade7-9	Adolescents	Bandura original	Individualism	61.4
Zhou et al., 2019	655	0.440	17–26	Adults	Bandura revision	Collectivism	52.98
Jung and Park, 2020	551	0.260	Secondary1-3	Adolescents	Bandura revision	Collectivism	50.5
Jung and Park, 2020	551	0.280	Secondary1-3	Adolescents	Bandura revision	Collectivism	50.5
Pornari, and Pornari, 2010	334	0.290	13.3±0.9	Adolescents	Bandura original	Individualism	53.1
Moses, 2013	390	0.388	Freshman	Adults	Bandura original	Individualism	59
Almeida et al., 2012	499	0.280	11–18	Adolescents	Bandura original	Collectivism	47.1
Wang and Ngai, 2020	1,103	0.309	15.3±1.576	Adolescents	Bandura revision	Collectivism	52.5
Wang et al., 2020	2,393	0.46	12.75±0.58	Adolescents	Bandura original	Collectivism	50.23
Lyu and Zhang, 2017	187	0.43	17–23	Adults	Bandura revision	Collectivism	48.67
Gao et al., 2020	2,393	0.44	12.75±0.58	Adolescents	Bandura original	Collectivism	50.23
Marín-López et al., 2020	1,033	0.35	13.66±1.64	Adolescents	Others	Collectivism	48.32

(1) *N*=the number of participants included in the present meta-analyses; (2) *MD* Measure is a measure of moral disengagement; (3) Bandura original is a Bandura’s original moral disengagement questionnaire, and Bandura revision is a revised version of Bandura moral disengagement questionnaire; (4) add a and b after the publication year to code, a,b Samples were the first author publishes two papers in the same year; and (5) if the same research includes two independent samples, add 1, 2 after the first author to make a distinction, 1, 2 Samples from the same study. (6) Collectivism=Collectivism culture, Individualism=Individualism culture.

#### Sample Characteristics

Two types of sample characteristics were coded in the present study: (1) gender was coded according to the percentage of girls included in the sample; (2) age was coded for adolescents (range of mean age: 6–18years old) and adults (range of mean age: 18years old above). These were coded to examine whether the strength of the association between moral disengagement and cyberbullying varied across the participant samples.

#### Study Design and Outcome Characteristics

Regarding the study design and outcome characteristics, the relationship between moral disengagement and cyberbullying was first examined in the present study. Then, the moderating variable was coded. Considering that there are fewer studies on the cultural background between moral disengagement and cyberbullying, classification in the present study was made using Hofstede’s cultural model according to collectivism and individualism. The cultural background was divided into collectivism and individualism. Previous studies have adopted various scales to examine the relationship between moral disengagement and cyberbullying. The classification of the Moral Disagreement Scale was divided into three main categories (Bandura Original, Bandura Revised, and Others). The original Bandura scale was the initial scale developed by Bandura et al. (1996), and the revised Bandura scale was developed for different countries and regions with the promotion and revision of the scale. The other scales were self-compiled by other researchers, and all the scales possessed good reliability and validity. Thus, three different categories of moral disengagement tools were employed in the present study (Bandura Original vs. Bandura Revised vs. Others). Furthermore, the age was divided into adolescent and adult. In addition to the three classified variables (age, moral disengagement tools, and cultural background), the proportion of women was adopted as a moderating variable, and gender was coded as a continuous variable.

### Data Extraction

The two authors coded documents simultaneously, and the coding process was completed independently without communication. After the independent coding, two researchers compared the coding documents and cross-checked the results. The identical coding rate was 97.2. For the differences in the screening and data extraction processes between the two authors, they addressed the problem and finished the final code documents. All authors strictly adhered to the inclusion criteria guidelines.

### Meta-Analytic Procedure

The meta-analysis strategy used comprehensive meta-analysis CMA 3.0 (CMA; Higgins and Green, 2005; Borenstein et al., 2013; Higgins et al., 2019). In the primary analysis, the overall effect size was represented by *r* to make the report clearer. Cohen (1992) concluded that the effect size *r*=0.10 is small, *r*=0.30 is medium, and *r*=0.50 is large. These guidelines are employed to assess the effect size of relationships reported in the meta-analysis. The Pearson correlation coefficient *r* is taken as the effect size of the relationship between moral disengagement and cyberbullying because it is easy for Pearson *r* to explain the effect size indicator of the relationship between the variables. If Pearson *r* was not reported, the effect size *r* should be calculated by other available data in the study. CMA converted all effect sizes *r* (Hedges and Olkin, 1985) to calculate the combined effect size. Then, the *Q* statistics (statistical testing of heterogeneity) and the *I*^2^ index (representing the amount of heterogeneity) were used to evaluate the effect sizes in each study (Higgins et al., 2019). The range of *I*^2^ values was 0–100%, of which 25% represents low heterogeneity, 50% represents medium intensity heterogeneity, and 75% represents high heterogeneity (Higgins et al., 2003).

Furthermore, a moderator analysis (measuring tools, participant age, gender, and cultural background) was conducted, and the level of influence of each moderating factor was estimated in this study. All analyses adopted the random-effects model (Hedges and Vevea, 1998). The random-effects model was selected to integrate the effect size to reduce the chance of the Type *I* error (Borenstein et al., 2010). By convention, the criterion for statistical significance was usually set as a value of *p* less than 0.05 (Borenstein et al., 2009), and data on the 95% confidence interval of the effect size were given. Publication bias indicates whether the published research literature can systematically and comprehensively represent the research population in this field (Rothstein et al., 2005). In the study, the *Q* test was performed to test the heterogeneity of the data, and three test methods (Funnel plot, Fail-safe Number (*N_fs_*), and *Egger’s* regression intercept method) were used to test whether the publication bias exists.

## Results

### Study Characteristics

After excluding studies according to our predefined criteria, a total of 32 articles, 38 effect sizes (*N*=38,425 participants) were included in the analyses (Figure 1 was a flow chart depicting reasons for article exclusions). An overview of all included studies is presented in Table 2. We have excluded some articles following the inclusion criteria, and all articles were peer-reviewed publications. The primary studies were conducted between 2005 and February 30, 2021. We could not include any earlier articles. Owing to the popularity of the Internet, the concept of cyberbullying only appeared after 2005. Most studies were performed in collectivist culture countries (*k*=24), and some were conducted in individualistic culture countries (*k*=14). The participants of the samples were adolescents (*k*=33) and adults (*k*=5). Moreover, all studies had correlational studies (*k*=38), with no experimental designs.

### Effect Size and Homogeneity Tests

The random-effects model was used for the test of the main effects (as shown in Table 3). The overall correlation coefficient between moral disengagement and cyberbullying was 0.341 (*p*<0.001). According to the criteria above, the relationship between moral disengagement and cyberbullying was a medium correlation in magnitude. Most effect sizes ranged between 0 and 0.5. As revealed from Table 2, the *Q*-value of the effective value of the relationship between moral disengagement and cyberbullying reached a significant level after the heterogeneity test (*p*<0.001), indicating that the effect size in the meta-analysis was heterogeneous. The *I*^2^ value was 95.351 (Table 3). It suggested that the effect size of moral disengagement and cyberbullying was highly heterogeneous. Thus, a random effect model should be used for analysis in this study.

**Table 3:** Summary and the moderating effect test between moral disengagement and cyberbullying.

	***Q***_***b***_	Value of *p*	*k*	*N*	*r*	*95% CI*		Two-tailed test	
						*LL*	*UL*	*Z*	*p*
**Overall**			38	38,425	0.341	0.291	0.389	12.483	0.000
***Age***	5.532	0.019	38						
adult			5	6,901	0.478	0.356	0.584	6.901	0.000
adolescent			33	31,524	0.319	0.267	0.369	11.357	0.000
***Culture background***	5.792	0.016							
Collectivism			24	29,905	0.380	0.327	0.431	12.890	0.000
Individualism			14	8,520	0.270	0.194	0.342	6.786	0.000
***MD measure***	1.429	0.489							
Bandur original			22	29,129	0.339	0.272	0.403	9.345	0.000
Bandur revision			12	6,828	0.369	0.279	0.452	7.572	0.000
Others			4	2,468	0.258	0.089	0.413	2.952	0.003
***Female (%)***	80.330	0.000							
			38	38,425	0.342	0.289	0.392	11.873	0.000

*k*=number of samples; *N*=number of participants; *r*=sample-size-summary observed validity; *CI*=confidence interval of r; *LL*=lower limit; *UL*=upper limit; *Z*=*z*-test values for the mean differences between the corrected correlation in a row and the corrected correlations in each of the following rows within that moderation category.

### Publication Bias

**Figure 2:**
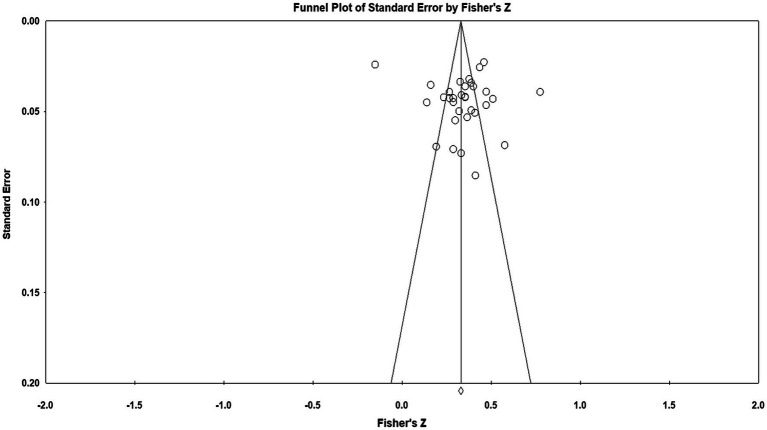
The funnel plots are in this meta-analysis.

First, a funnel plot was performed to measure the publication bias of this meta-analysis, as exhibited in Figure 2. The funnel plot demonstrated that the research literature on moral disengagement and cyberbullying was uniformly and symmetrically distributed on two sides of the total effect size, and most of the research data were mainly concentrated in the middle and upper part of the funnel plot, reflecting that there was a little possibility of publication bias in this meta-analysis. However, the funnel plot was only used for the preliminary examination of publication bias from an intuitive perspective, and Rosenthal’s Classic Fail-Safe *N* and *Egger’s* regression intercept method were employed to perform a more accurate inspection. According to Rosenthal, the Fail-Safe *N* factor was greater than 5*k*+10 (*k* is the number of studies), indicating that the meta-analysis publication bias was effectively controlled (Rothstein et al., 2005). *Egger’s* regression intercept method is usually performed in a hypothesis test on whether the intercept is 0. If it is not significant, there is no publication bias (Egger et al., 1997). In this study, the Fail-Safe *N* coefficient (*N_fs_*) of cyberbullying was 30,579, which was much larger than 5*k*+10=200, suggesting no publication bias. Meanwhile, *Egger’s* regression showed that the intercept value was 2.246 (*p*>0.05), further confirming that there was no publication bias in this study. Therefore, the published research literature included in this study can systematically and comprehensively represent the research population in this field.

### Moderator Analyses

Four moderator analyses were conducted, respectively, for the sample age group (Adults vs. Adolescents), moral disengagement measure tools (Bandura original vs. Bandura revision vs. Others), cultural background (Collectivism culture vs. Individualism culture), and gender (Proportion of women).

Before our meta-analyses, a qualitative and quantitative review of these four moderator variables was performed. The heterogeneity test results suggested that the overall effect size of the included literature was highly heterogeneous, demonstrating there had a significant moderating effect (Table 3). Regarding measuring tools, there was no significant moderating effect. The MDS types presented no moderating effect on the relationship between moral disengagement and cyberbullying (*Qb*=1.429, *p*>0.05). Given the previous analysis, the MDS was divided into three categories. However, different MDSs all exhibited a good Coefficient of Internal Consistency. Therefore, different MDSs may not have an impact on the relationship between moral disengagement and cyberbullying. The participant’s age had a significant moderating effect on the relationship between moral disengagement and cyberbullying (*Qb*=5.532, *p*=0.019), with the effect size of the adult group (*r*=0.488) significantly higher than that of the adolescent group (*r*=0.308). Gender had a significant moderating effect on the relationship between moral disengagement and cyberbullying (*Qb*=80.330, *p*=0.000). As the proportion of female subjects in the study increases, the effect size increases. The cultural background had a significant moderating effect on the relationship between moral disengagement and cyberbullying (*Qb*=5.792, *p*=0.016), with the effect size of the collectivist culture (*r*=0.380) higher than that of the individualism culture (*r*=0.270).

## Discussion

In this study, a meta-analysis was conducted to quantitatively summarize the empirical research on the relationship between moral disengagement and cyberbullying. The results suggested that moral disengagement and cyberbullying had a positive correlation of medium intensity, implying that cyberbullying behaviors will be more frequent for individuals with higher moral disengagement.

Individuals with morally disengaged lack self-censorship are more callous and tend to engage in cyberbullying (Fang et al., 2020). The reason why some students cyberbully is just that they are bored, looking for fun, and entertaining themselves (Kyriacou and Soteriou, 2015; Ramadan, 2019). Bandura (2002) argued that the process of moral disengagement centers on redefining harmful conduct as honorable by moral justification. It focuses on the agency of action, enabling the perpetrators to minimize their role in causing harm by diffusion and displacement of responsibility, to minimize or distort the harm resulting from detrimental actions. This makes the group of cyberbullying easier to escape responsibility in a moral disengagement (Meter and Bauman, 2016). In the online world, perpetrators of cyberbullying often perceive themselves to be anonymous. Individuals will say and do things anonymously, rather than in face-to-face interactions. The anonymity of the network significantly opens up the pool of potential perpetrators of cyberbullying. Cyberbullying information is often released by nicknames person, making it impossible to identify and find cyberbullies quickly. It is easy to use the moral disengagement mechanisms to generate cyberbullying, leaving victims in a passive position and unable to effectively combat cyberbullying (Wang et al., 2016, 2020). In traditional bullying, bullying often happens face to face. When the victim is separated from the bully, it can prevent being bullied and reduce the harm caused by the bullying behavior to the victim (Bussey et al., 2015a). However, cyberbullying, which is not restricted by time and space, can happen anytime and anywhere (Lazuras et al., 2019; Hoareau et al., 2020). The pictures, videos, and text messages of cyberbullying others posted on the Internet are permanent. This information will not disappear with time or memory degradation. Moreover, it is also easier to cause the spread of cyberbullying information with the convenience and popularization of functions, such as “sharing” and “reposting.” People “sharing” and “reposting” information without discriminating between right and wrong in cyberspace are a secondary injury to victims (Selkie et al., 2015; Chan et al., 2021). When cyberbullying becomes a collective activity, everyone’s responsibilities are reduced. The disengagement of collective morality also encourages cyberbullying, causing more harm to the victims.

For the measuring tools, there was no moderating effect on the relationship between moral disengagement and cyberbullying. The measurement of moral disengagement had no significant effect on the relationship between moral disengagement and cyberbullying. Related research demonstrated that the differences in MDSs are caused by common method bias (Gini et al., 2014a; Kowalski et al., 2018). In recent years, with the deepening of research and the awakening of people’s moral consciousness, different MDSs have been continuously revised in the research (Gini et al., 2014a). Current MDSs generally have good content validity and structural validity, and the scales used possess good reliability and validity (Wang et al., 2014; Zhou et al., 2019). The dimensions of the MDSs adapted by researchers in different countries were similar (Kowalski et al., 2014). Consequently, there was no moderating effect on the relationship between moral disengagement and cyberbullying.

We also discovered that age has significantly moderated the relationship between moral disengagement and cyberbullying. This relationship was higher among adults than among adolescents. Compared to younger students, adults were more likely to engage in moral disengagement and cyberbullying (Gini et al., 2014a; Kowalski et al., 2018). Some researchers revealed that adults and college students, without academic pressure and parental supervision, had more access to social media and spent more time online than teenagers (Berger, 2007; Kowalski et al., 2014; Chen et al., 2016). Thus, they were more likely to be morally disengaged without supervision, resulting in more cyberbullying (Kowalski et al., 2014; Martinez-Pecino and Duran, 2019; Marr and Duell, 2020). Some researchers pointed out that since adults acquired more skills in using the Internet, they could adopt various ways to bully others on the Internet. More adults may also realize that cyberbullying is more accessible and safer than direct bullying. Therefore, they are more likely to morally disengage in the online environment and engage in cyberbullying (Chen et al., 2016). Furthermore, as they grow older, bullies begin to realize that their direct bullying behavior is not in accordance with social norms, and they need to pay a certain price or be severely punished (Kowalski et al., 2018; Chan et al., 2021). Thus, individuals tend to use indirect forms of bullying to maintain their image and avoid punishment. Cyberbullying is a kind of indirect bullying behavior (Gini et al., 2014a). Its anonymity and concealment are more conducive to the moral disengagement of elderly bullies and enhance their cyberbullying behavior (Zych et al., 2019a). With the growth of age, the number of cyberbullies increased.

Gender had a significant moderating effect on the relationship between moral disengagement and cyberbullying. As the proportion of women increased, the correlation coefficient between moral disengagement and cyberbullying increased. This relationship is higher among females than among males. Females prefer indirect bullying while males tend to direct bullying (Alhajji et al., 2019). Cyberbullying is an indirect form of bullying. Cyberbullying does not require face-to-face contact, and its invisibility may attract girls’ “hidden aggression culture” (Busching and Krahé, 2015; Pereira and Matos, 2016). Particularly, their moral disengagement mechanisms are activated in cyberspace. Girls who cyberbully may hide behind a mask of anonymity. They try to intimidate those who are physically stronger than they are, or who have more advantages than they have, or who are unable to compete with them in real life, leading to cyberbullying (Kowalski et al., 2014; Calmaestra et al., 2020; Gao et al., 2020). Meanwhile, some traditional bullying victims may use the Internet to attack others in retaliation. Some cyberbullying girls whose power in the real society is weak were likely to be victims of traditional bullying (Raskauskas et al., 2010; Robson and Witenberg, 2013). Then, they vented emotions by attacking others on the Internet. The uniqueness of the Internet and the anonymity of the Internet increased the activation of the moral disengagement mechanism and protected girls’ cyberbullying behavior, resulting in more cyberbullying (Marr and Duell, 2020). Moreover, many researchers considered that women use emerging digital communication platforms more than men, making them more likely to develop unhappy relationships online. Hence, the risk of online quarrels and conflicts increases, leading to more moral disengagement and cyberbullying (Marr and Duell, 2020).

Moreover, the correlation coefficient between moral disengagement and cyberbullying under the background of collectivism was higher than that in individualism, according to Hofstede’s cross-cultural model. In a collectivist culture, the behavior of an individual was often dependent on and inseparable from the collective behavior (Wang et al., 2019b). In collective behavior, it is more conducive for individuals to make moral justification for their immoral behaviors, blur and distort their immoral behaviors, and attribute their faults to others (Allison and Bussey, 2016; Li et al., 2021). In this way, they can evade responsibility and transfer responsibility, reducing everyone’s sense of responsibility and responsible attitude toward cyberbullying (Gini et al., 2014b, 2015). In a collectivist environment, it is easy for individuals to produce moral disengagement mechanisms. When the group engages in cyberbullying, the individual may also provide help to the collective behavior under the influence of the group, such as group fighting (Allison and Bussey, 2017). The higher the possibility of moral disengagement with anonymity in a network environment, the more likely it is to increase the individual’s cyberbullying behavior (Fernández-Antelo and Cuadrado-Gordillo, 2019; Fang et al., 2020). However, individuals cannot get collective support and often have a sense of insecurity in the individualistic culture. Thus, it is not easy to activate the moral disengagement mechanism, weakening the occurrence of cyberbullying behavior (Bjärehed et al., 2019, 2021). Thus, cyberbullies tend to be fewer in individualistic cultures.

### Contributions

This systematic review offered three crucial contributions. First, the effect size between moral disengagement and cyberbullying was explored. This provided a further study of cyberbullying for academic literature. Moreover, several moderators of these relationships were tested to illuminate further the effect size of the moderating variable on the relationship between moral disengagement and cyberbullying. Anonymity, invisibility, and disinhibition in the network environment were more likely to be the mechanisms inducing moral disengagement and cyberbullying.

Second, cross-cultural research was performed on the relationship between moral disengagement and cyberbullying, and discovered that the relationship between moral disengagement and cyberbullying in the collectivist cultural background was higher than that in the individualism cultural background. In the collectivist culture, the actions of individuals were often influenced by the group. The indirect network environment has induced more moral disengagement mechanisms, leading to conformity and imitation in the groups and cyberbullying behavior. Moreover, individual behavior was often hard to be affected by collective behavior in the cultural context of individualism.

Third, the influence of participants’ characteristics on the relationship between moral disengagement and cyberbullying was researched. Specifically, gender had a moderating effect on the relationship between moral disengagement and cyberbullying. This filled up the blanks in past research. In the previous studies, the role of gender as a demographic variable in cyberbullying behavior was controversial. In this study, the method of female ratio was adopted to reveal that the effect size between moral disengagement and cyberbullying increases as the proportion of women increases. Women’s unique characteristics and hidden network atmosphere give birth to women’s cyberbullying behavior. Next, age had significantly moderated the relationship between moral disengagement and cyberbullying. The effect size of adults was significantly higher than that of adolescents. The main reason for this phenomenon is that adults have more time to use the Internet and master more Internet skills, allowing them to use the Internet more to engage in cyberbullying.

### Limitations and Future Directions

(1) In this study, eligible meta-analysis articles were screened and included. However, some conference papers and dissertations were still not available due to copyright restrictions, causing a small amount of data to be omitted. Some papers have no direct reporting effect size. Since the method of transformation was adopted to include the effect size, there may be a certain error. Therefore, the search for the original data in the article should be expanded in future research.

(2) Some studies pointed out that longitudinal researches should be designed to examine the gender correlation between moral disengagement and cyberbullying, and gender-atypical samples cannot be included in the analysis (Navarro et al., 2016). Gender typicality has been commonly used as an indicator of participants’ conformity to gender congruent attributes and traits (Jackson et al., 2020). Gender typicality is the self-perceived similarity to other members of the same gender category that is more abstracted and synthesizes diverse information about one’s gender typing (Navarro et al., 2016). Gender-atypical is the opposite of gender typicality. For example, atypical sex boys, who prefer to be more like girls in some aspects, such as personality traits, activity preferences, academic pursuits, and occupational preferences. Future studies of cyberbullying should consider including measures of gender typicality and gender identity to more fully account for gender effects (Perry et al., 2019; Jackson and Bussey, 2020).

(3) Apart from cross-sectional study on moral disengagement and cyberbullying, longitudinal research on their relationship can also be increased. Personal experience, years of network usage, and parental rearing styles may also have an impact on moral disengagement and cyberbullying. These variables can be studied in future research. By tracking the relationship between moral disengagement and cyberbullying, as men and women grow older, we can determine who is more aggressive in different circumstances, girls or boys (Leduc et al., 2018). Furthermore, experimental studies will be designed to verify the theory of causality with moral disengagement as the independent variable and cyberbullying as the dependent variable, so as to determine whether moral disengagement is a necessary factor of cyberbullying.

(4) At present, there are not sufficient studies to confirm the impact of the dimensionality mechanism of moral disengagement on cyberbullying, and future studies need further subdivide the relationship between specific moral disengagement mechanisms and cyberbullying. (5) Due to the limitations of the sample age range, the moderating effect across the age range of our samples in this study does not mean that cyberbullying is directly proportional to the increase of age, which requires the inclusion of older subjects and more in-depth studies in further research. (6) Most of the existing research data were mostly self-reporting methods, which may be affected by the social desirability effect (Wang et al., 2016) and increase the overall effect size. In future research, a multi-angle cyberbullying report method (including parents, friends, and teachers) should be employed (Calvete et al., 2010; Beran et al., 2012).

## Conclusion

(1) There was a medium positive correlation between moral disengagement and cyberbullying. (2) The measuring tools did not have a moderating effect on the relationship between moral disengagement and cyberbullying behavior. (3) Age played a significant role in moderating the relationship between moral disengagement and cyberbullying. The effect size between moral disengagement and cyberbullying in the adult group was significantly higher than that in the adolescent group. (4) Gender played a significant role in moderating the relationship between moral disengagement and cyberbullying. The correlation coefficient between moral disengagement and cyberbullying increased with the increase in the proportion of women in the sample. (5) Cultural background had a significant moderating effect on the relationship between moral disengagement and cyberbullying, and the correlation coefficient between moral disengagement and cyberbullying in the collectivist cultural background was higher than that in the individualism cultural background.

## Data Availability Statement

The original contributions presented in the study are included in the article/supplementary material, further inquiries can be directed to the corresponding author.

## Author Contributions

LZ designed the study and wrote the protocol. JY conducted the statistical analysis and wrote the first draft of the manuscript. All authors contributed to the article and approved the final manuscript. All authors made equal contributions to the final version for submission.

## Funding

This research was supported by the Social Science Planning Research Project of Shandong Province, China (Grant No. 17CZLJ03), and the Postgraduate Education and Teaching Reform Research Project of Shandong Province, China (Grant No. sdyjg21200).

## Conflict of Interest

The authors declare that the research was conducted in the absence of any commercial or financial relationships that could be construed as a potential conflict of interest.

## Publisher’s Note

All claims expressed in this article are solely those of the authors and do not necessarily represent those of their affiliated organizations, or those of the publisher, the editors and the reviewers. Any product that may be evaluated in this article, or claim that may be made by its manufacturer, is not guaranteed or endorsed by the publisher.
